# Potential usefulness of ^68^Ga-citrate PET/CT in detecting infected lower limb prostheses

**DOI:** 10.1186/s13550-018-0468-3

**Published:** 2019-01-03

**Authors:** Jing-Ren Tseng, Yu-Han Chang, Lan-Yan Yang, Chen-Te Wu, Szu-Yuan Chen, Chih-Hsing Wan, Ing-Tsung Hsiao, Tzu-Chen Yen

**Affiliations:** 1 Department of Nuclear Medicine and Center for Advanced Molecular Imaging and Translation, Chang Gung Memorial Hospital at Linkou, No. 5, Fu-Hsing ST., Kwei-Shan, Taoyuan, Taiwan; 2 Department of Medical Imaging and Radiological Science and Healthy Aging Center, College of Medicine, Chang Gung University, Taoyuan, Taiwan; 3 Bone and Joint Research Center and Department of Orthopaedic Surgery, Chang Gung Memorial Hospital at Linkou, Taoyuan, Taiwan; 4 Biostatistics Unit, Clinical Trial Center, Chang Gung Memorial Hospital at Linkou, Taoyuan, Taiwan; 5 Department of Medical Imaging and Intervention, Chang Gung Memorial Hospital at Linkou, Taoyuan, Taiwan; 6 Department of Nuclear Medicine, Mackay Memorial Hospital, Taipei, Taiwan

**Keywords:** ^68^Ga-citrate, ^18^F-FDG, PET/CT, Prosthetic joint infections

## Abstract

**Background:**

Prosthetic joint infections may lead to failures of total joint arthroplasty. Radionuclide imaging can play a diagnostic role in identifying such infections, which require two-stage exchange arthroplasty (instead of simple revision surgery performed in non-infected cases). Although ^18^F-FDG PET/CT has emerged as a novel diagnostic tool in this setting, the clinical usefulness of ^68^Ga-citrate PET/CT has not been previously investigated. This single-center prospective study was designed to address this issue.

**Methods:**

Between January 2016 and October 2017, we examined 34 patients with clinically proven or suspected prosthetic hip/knee joint infections scheduled to undergo surgery. All patients underwent ^68^Ga-citrate PET/CT scans and sequential ^18^F-FDG PET/CT imaging for comparative purposes. Intraoperative findings and the results of microbiological analyses of surgical specimens served as gold standard. The diagnostic results were examined according to (1) image interpretation based on radiotracer uptake patterns and (2) quantitative analysis using volumes of interest (VOIs) to calculate standard uptake values (SUVs) and metabolic volumes (MVs).

**Results:**

A total of 26 (76%) patients were diagnosed as having infections. Based on radiotracer uptake pattern criteria, the sensitivity, specificity, and accuracy of ^68^Ga-citrate PET/CT and ^18^F-FDG PET/CT were 92%, 88%, and 91% and 100%, 38%, and 85%, respectively. MV was significantly higher in the infected group when ^68^Ga-citrate PET/CT was used (422.45 vs. 303.65 cm^3^, *p* = 0.027), whereas no significant differences were observed on ^18^F-FDG PET/CT. According to receiver operating characteristic (ROC) curve analysis, a cut-off value of 370.86 for MV resulted in a sensitivity of 61.5% and a specificity of 87.5% (area under curve: 0.75, 95% confidence interval: 0.57–0.88, *p* = 0.035).

**Conclusions:**

Subject to future confirmation, our data provide preliminary evidence that ^68^Ga-citrate PET/CT may have a complimentary role to ^18^F-FDG PET/CT in detecting prosthetic joint infections, being characterized by a higher specificity and the possibility to discriminate between an infectious condition and sterile inflammation.

**Trial registration:**

This prospective study was registered at clinicaltrials.gov (registration number: NCT02855190).

## Background

Total joint arthroplasty is one of most frequently performed and successful surgical procedures in orthopedics [1]. However, between 0.4 and 4% of all joint replacement procedures ultimately develop infectious complications, with a 2–18% of cases showing aseptic loosening [2]. Numerous conventional nuclear medicine techniques have been investigated as imaging tools to discriminate between an infectious condition and sterile inflammation—including technetium-99m methylene diphosphonate (MDP) bone scintigraphy (BS), gallium (Ga)-67 citrate scans, and indium-111 or technetium-99m hexamethylpropylene amine oxime (HMPAO)-labeled white blood cell (WBC) scans [3]. However, each of these techniques has significant limitations, including (1) the lack of specificity typical of BS and Ga-67 citrate scans, (2) the suboptimal imaging characteristics of all conventional two-dimensional examinations, (3) the potentially hazardous preparation of radiopharmaceuticals required by WBC scans, (4) the prolonged physical and biological half-life of indium-111- or Ga-67-labeled agents (resulting in a high absorbed radiation dose), and (5) the need of combining different traditional scanning techniques to obtain reliable diagnostic results. All of these caveats ultimately prevent their routine clinical use [4–7].

^18^F-fluoro-2-deoxyglucose positron emission tomography/computed tomography (^18^F-FDG PET/CT) is increasingly emerging as a useful diagnostic tool for several infectious and inflammatory conditions—especially in patients with chronic renal failure or in those who had undergone metal prosthesis implantation [8, 9]. Previous studies have shown that ^18^F-FDG PET imaging has a pooled sensitivity of 82.1–82.8% and a specificity of 86.6–87.3% for the diagnosis of lower limb prosthetic joint infections [10, 11]. In this setting, ^18^F-FDG PET imaging may also offer significant advantages over conventional nuclear medicine examinations (which not only are more complex and expensive but also potentially limited by safety issues) [12]. ^68^Gallium (^68^Ga) is a positron-emitting isotope that has been previously used for PET imaging. Intriguingly, preliminary data on the potential usefulness of ^68^Ga-citrate PET for identifying patients with bone infections have been promising [13]. In a head-to-head comparison, ^18^F-FDG PET/CT has been superior to ^68^Ga-citrate PET/CT for diagnosing inflammatory reactions elicited by metal debris [14]. However, the question as to whether ^68^Ga-citrate PET/CT is either superior or may offer complimentary information to ^18^F-FDG PET/CT in detecting lower limb prosthesis infections remains open. This prospective study was designed to address this research question.

## Methods

### Ethics statement

This study had a prospective design. The protocol complied with the tenets of the Declaration of Helsinki, was approved by the Institutional Review Board of the Chang Gung Memorial Hospital (CGMH) at Linkou (approval number: 103-7266A), and was registered at clinicaltrials.gov (NCT02855190). All patients gave their written informed consent. All data were securely protected (by delinking personal information from the main data sets), made available to investigators only, and analyzed anonymously.

### ^68^Ga-citrate synthesis

^68^Ga was obtained using a ^68^Ge/^68^Ga generator (ITG Isotope Technologies Garching GmbH, Garching, Germany) which was eluted with 2.5 ml of 0.05 mol/l hydrogen chloride (HCl) solution into a 20-ml sterile vial—which contained 2.5 ml of sterile sodium citrate (27 mg/ml) solution, and 5 ml of sterile injection water. The ^68^Ga sodium citrate solution was subsequently transferred into the product vial through a sterile filter. The radiochemical purity of ^68^Ga-citrate was analyzed with an instant thin layer chromatography-silica-gel technique using methanol/acetic acid (9:1) as the mobile phase. The product pH was tested using indicator strips (pH range, 4.0–10.0), whereas the integrity of sterile filters was assessed with a bubble point test. The ^68^Ga-citrate used in this study was characterized by (1) high radiochemical purity (≥ 97%), (2) a pH of 4.5–8.0, and (3) a strength ≥ 11.1 MBq/ml.

### Patient selection

Between January 2016 and October 2017, a total of 39 patients were deemed eligible for the study. Inclusion criteria were as follows: (1) clinically proven or suspected periprosthetic hip/knee joint infections and (2) scheduled surgery. Patients were excluded if they met one of the following criteria: (1) pregnancy or breastfeeding, (2) significantly abnormal laboratory findings, and (3) critical illnesses or unstable vital signs that made the patient unsuitable for imaging and surgical work-ups. Four patients were excluded during the screening period, whereas one additional patient who completed the requested imaging studies did not ultimately undergo surgery. After these exclusions, 34 patients were retained in the analysis. All of the study participants were followed up for at least 6 months.

### PET/CT acquisition and processing

All of the patients underwent sequential ^68^Ga-citrate PET/CT and ^18^F-FDG PET/CT imaging performed within less than 1 week of each other. The order of the two scans was not predetermined. The injection dose was 111–185 MBq for ^68^Ga-citrate and 5.18 MBq per kilogram of body weight for ^18^F-FDG. Patients were required to fast for at least 4 h before injection of both radiotracers. Images were obtained 60 min after intravenous administration of each tracer via a Biograph mCT PET/CT system (Siemens Healthineers, Erlangen, Germany). Patients were scanned from the iliac crest to the toe. CT scanning parameters for ^68^Ga-citrate and ^18^F-FDG examinations were 120 kV; 0.5 s per rotation; collimation, 40 × 0.6; pitch, 1.5; quality reference mAs with CAREDose 4D, 100 mAs; and slice thickness, 5 mm. PET acquisition time per bed position was 3 min for ^68^Ga-citrate and 1.5 min for ^18^F-FDG. All of the images were reconstructed using ultra-HD reconstruction with time of flight (2 iterations and 21 subsets), a 200 × 200 image matrix, and a 3-mm full width at half maximum (FWHM) Gaussian filter. In all cases, image interpretation was performed before surgery.

### PET imaging interpretation by radiotracer uptake pattern

All of the PET images were CT attenuation-corrected. The criterion for defining an examination as positive was the presence of an abnormal radiotracer uptake located at the bone-prosthesis interface and/or periprosthetic soft tissue [10]. However, tracer uptake limited to soft tissues adjacent to the neck of the hip prosthesis was not considered sufficient to classify a scan as positive [15, 16]. Two observers (J.R.T. and T.C.Y.)—who were blinded to clinical data—jointly interpreted all images (including companion CT findings) and reached a diagnostic consensus through discussion. According to the definitions proposed by the Musculoskeletal Infection Society [17], the final diagnosis was based on intraoperative findings and microbiological evaluations of surgical specimens.

### PET imaging quantitative analysis

For each ^18^F-FDG and ^68^Ga-citrate image slide, a volume of interest (VOI) was manually drawn by an experienced nuclear medicine physician (J.R.T.) around the bone-prosthesis interface or periprosthetic soft tissue. To this aim, the PMOD 3.3 software package (PMOD Technologies Ltd., Zurich, Switzerland) was used. The standardized uptake value (SUV) was calculated as follows: SUV = (tissue radioactivity [Bq/ml])/(injected radioactivity [Bq]/body weight [g]). Maximum SUV (SUV_max_), mean SUV (SUV_mean_), and metabolic volume (MV) were subsequently calculated. The MV was defined as the number of voxel located inside the VOIs multiplied by voxel size. In this study, VOIs covered the entire bone-prosthesis interface as well as periprosthetic soft tissue (after the exclusion of vascular radioactivity). The isocontour was set at 20% of the maximum uptake observed within the corresponding focus.

### Statistical analysis

Based on an expected prevalence of confirmed infection of 75%, we initially planned to enroll 40 patients (resulting in a total of 30 patients with confirmed infection). However, recruitment was stopped at 39 cases because of a higher than expected incidence of cases with confirmed infections. The general characteristics of the study participants were summarized using descriptive statistics. Continuous data were expressed as medians and ranges and compared with the Mann-Whitney *U* test. Categorical variables were summarized as frequency counts and percentages. A 2 × 2 contingency table with four diagnostic outcomes (true positive, false positive, true negative, and false negative) was constructed based on the final diagnostic results. Receiver operating characteristic (ROC) curve analysis was applied to investigate the prediction accuracy. Optimal cut-off points that maximized prediction were identified using the Youden’s index. All calculations were performed using the SPSS software (version 22.0; IBM, Armonk, NY, USA). A two-tailed *p* value < 0.05 was considered statistically significant.

## Results

Table 1 shows the baseline characteristics of the study participants. Nineteen (56%) patients were male, and the median age was 64 years (range 31–86 years). Hypertension and diabetes were the most frequent comorbidities, accounting for 14 (41%) and 8 (23%) cases, respectively. Osteoarthritis was the most common underlying condition that required total joint arthroplasty. A total of 19 (56%) patients received hip arthroplasty, whereas 8 (23%) cases underwent contralateral prosthesis replacement as well. Five (15%) patients required a second debridement of the infected tissue during the follow-up period (median interval 60 days, range 20–120 days). The median interval between the last PET/CT scan and the collection of surgical specimens was 9 days (range 1–26 days). A total of 19 (57.6%) patients had a positive bacterial culture (*Staphylococcus aureus*, *n* = 6; *Staphylococcus epidermidis*, *n* = 4; and *Staphylococcus caprae*, *n* = 2). *Pseudomonas aeruginosa*, *Cellulosimicrobium cellulans*, *Enterococcus faecalis*, *Streptococcus dysgalactiae*, and *Staphylococcus lugdunensis* were isolated only in one case each. Two patients were positive for two or more bacteria. Specifically, one was positive for both *Staphylococcus aureus* and *Staphylococcus lugdunensis*, whereas the other was positive for four bacteria (*Staphylococcus aureus*, group B streptococcus, *Enterococcus faecalis*, and *Gemella morbillorum*).

**Table 1:** General characteristics of the study patients (*n* = 34)

	Entire cohort (*n* = 34)	Infected group (*n* = 26)	Non-infected group (*n* = 8)
Age, years	64 (31–86)	68.5 (31–86)	66 (44–67)
Male sex	19 (55.9%)	15 (57.7%)	4 (50%)
Clinical presentations			
Fever	1 (2.9%)	1 (3.8%)	0 (0%)
Joint pain	30 (88.2%)	22 (84.6%)	8 (100%)
Joint swelling	8 (23.5%)	8 (30.8%)	0 (0%)
Local heat	5 (14.7%)	5 (19.2%)	0 (0%)
Secretion	5 (14.7%)	5 (19.2%)	0 (0%)
Laboratory findings			
C-reactive protein, mg/L	21.7 (0.2–250.9)	35.7 (2–250.9)	1.4 (0.2–3)
White blood cells, 10^3^/μL	7.4 (1.8–18.0)	7.6 (1.8–18.0)	6.2 (4.4–9.1)
Erythrocyte sedimentation rate, mm/h	39 (2–116)	67 (2–116)	8 (18.5–36)
Cause of total joint arthroplasty			
Osteoarthritis	21 (61.8%)	18 (69.2%)	3 (37.5%)
Avascular necrosis	3 (8.8%)	2 (7.7%)	1 (12.5%)
Trauma	6 (17.6%)	4 (15.4%)	2 (25%)
Fracture	3 (8.8%)	2 (7.7%)	1 (12.5%)
Rheumatoid arthritis	1 (2.9%)	1 (3.8%)	0 (0%)
Prosthesis location, hip	19 (55.9%)	12 (46.2%)	7 (87.5%)
Prosthesis age, days	180 (7–6000)	180 (7–1800)	135 (58–6000)

Data are expressed as medians (ranges) for continuous variables. Categorical data are given as number and percentages

**Fig. 1:**
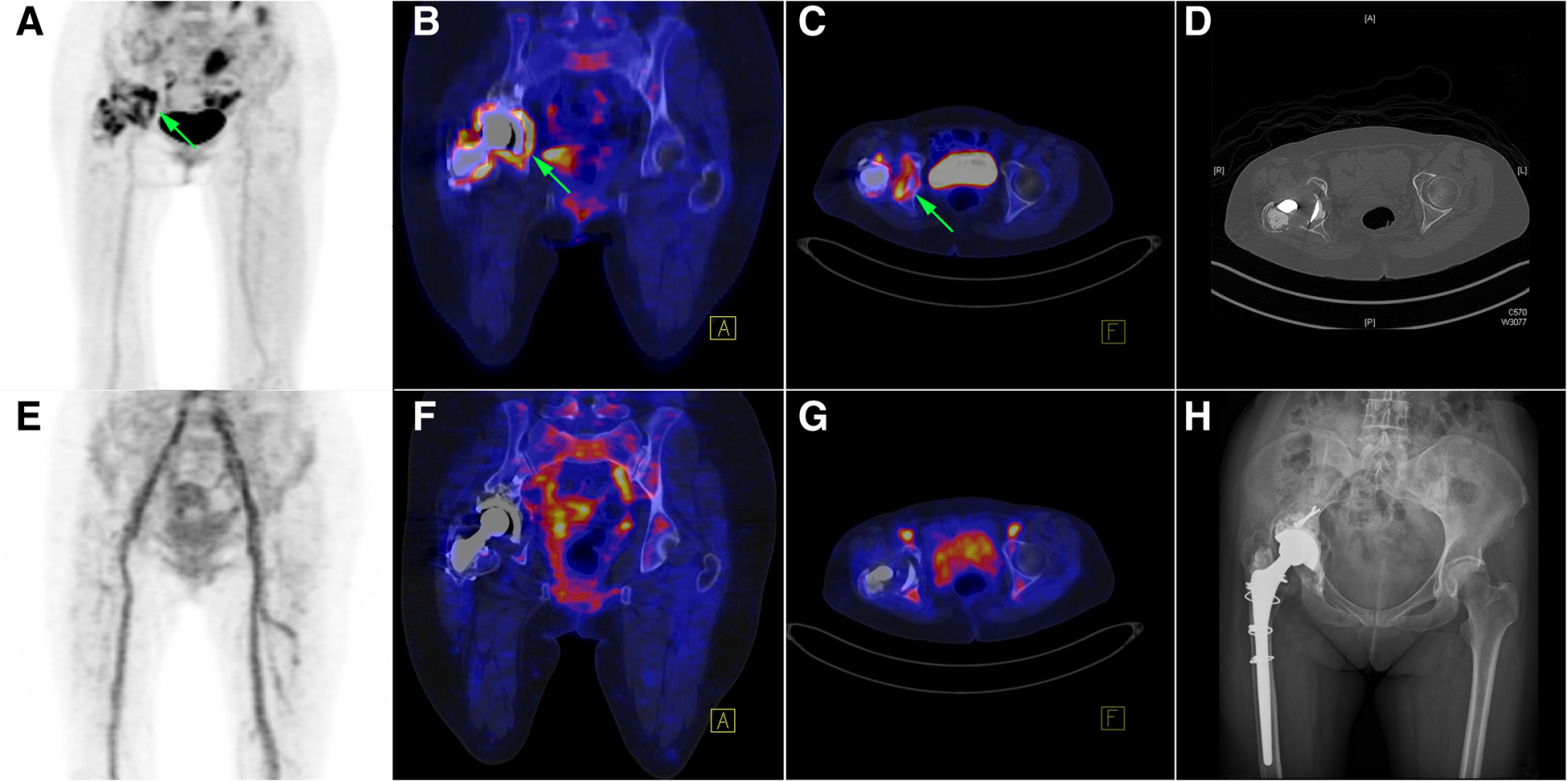
Patient (case # 16) with a history of right total hip arthroplasty performed 16 years before imaging. The results of ^18^F-FDG PET/CT (upper row) and ^68^Ga-citrate PET/CT (lower row) are presented. **a**–**d** Green arrows indicate an intense ^18^F-FDG uptake surrounding the cup and the proximal part of the hip prosthesis stem components. On CT images, osteolytic changes and the presence of residual cement in the right acetabulum were evident. Cup loosening with synovial hypertrophy and clear joint fluid were detected during surgery. ^18^F-FDG PET/CT findings were therefore classified as false positve. **d**–**f** Findings on ^68^Ga-citrate PET/CT were true negative, without any obvious radiotracer accumulation at the corresponding sites. **h** Plain film

**Fig. 2:**
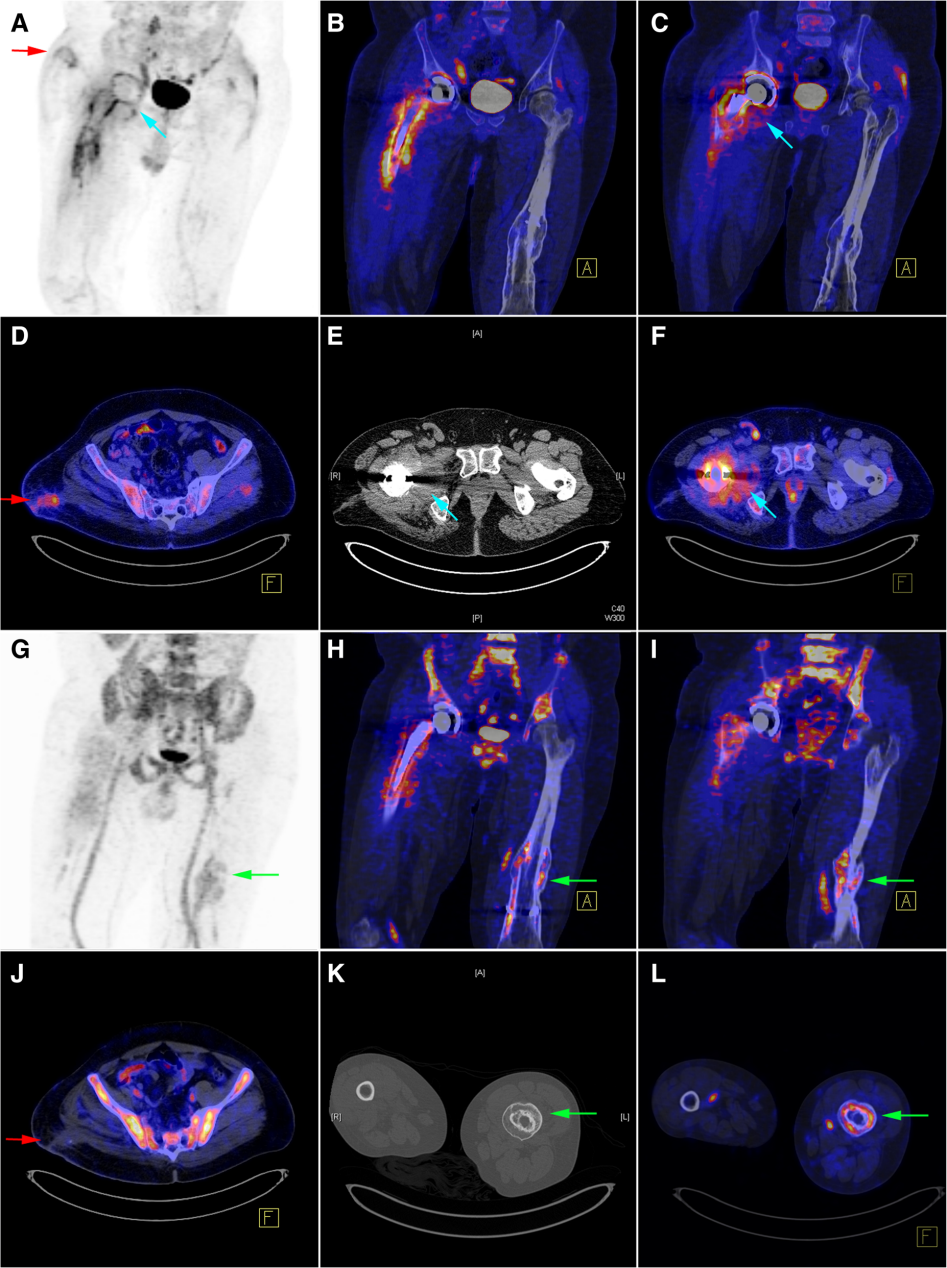
Patient (case # 1) with a history of right hip prosthesis implantation performed 7 months before imaging. The results of ^18^F-FDG PET/CT (upper two rows) and ^68^Ga-citrate PET/CT (lower two rows) are presented. **a**–**f** The red arrows clearly indicate ^18^F-FDG uptake occurring at the sinus tract, which was absent on the corresponding ^68^Ga-citrate PET/CT image. There was also evidence of swollen periarticular soft tissue (blue arrows) at the right ischiofemoral space on CT, which was accompanied by an increased ^18^F-FDG uptake. A similar pattern of radiotracer uptake (**b**, **h**) was found to occur at the bone-prosthesis interface of the stem component, as well as in the adjacent soft tissue. The imaging results were considered positive, and the case was subsequently classified as true positive. **g**–**l** The green arrows indicate an increased ^68^Ga-citrate uptake occurring at the left femoral shaft. The CT scan revealed cortical hypertrophy caused by a prior traumatic fracture followed by bone reunion

**Fig. 3:**
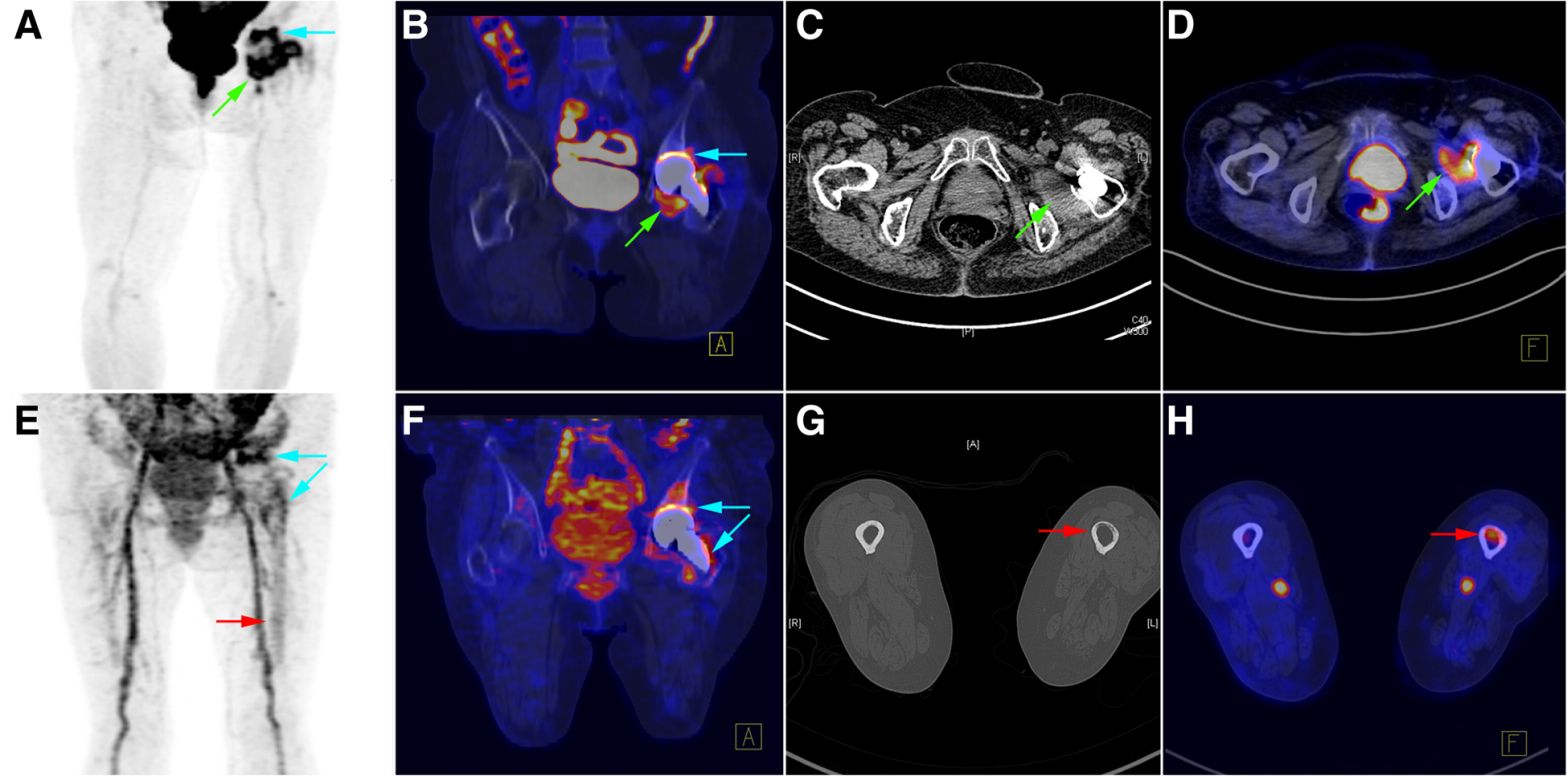
Patient (case # 14) with a history of hip prosthesis implantation performed 8 months before imaging. The results of ^18^F-FDG PET/CT (upper row) and ^68^Ga-citrate PET/CT (lower row) are presented. **a**–**d** The green arrows indicate an increased ^18^F-FDG uptake occurring in necrotic tissue located within a swollen left quadratus femoris muscle. There was also evidence of an increased ^18^F-FDG uptake occurring at the bone-prosthesis interface (cup component; blue arrow). Taken together, these findings were suggestive of an infectious process which was confirmed intraoperatively (presence of cloudy synovial fluid and necrotic tissue). **e**–**h** The blue arrows indicate an increased ^68^Ga-citrate uptake occurring at the bone-prosthesis interface (especially in the proximal portion). An osteolytic change in the anterior cortex of the left femur was observed on a CT image, accompanied by an increased ^68^Ga-citrate uptake (which was present in the adjacent bone marrow as well, red arrows)

**Fig. 4:**
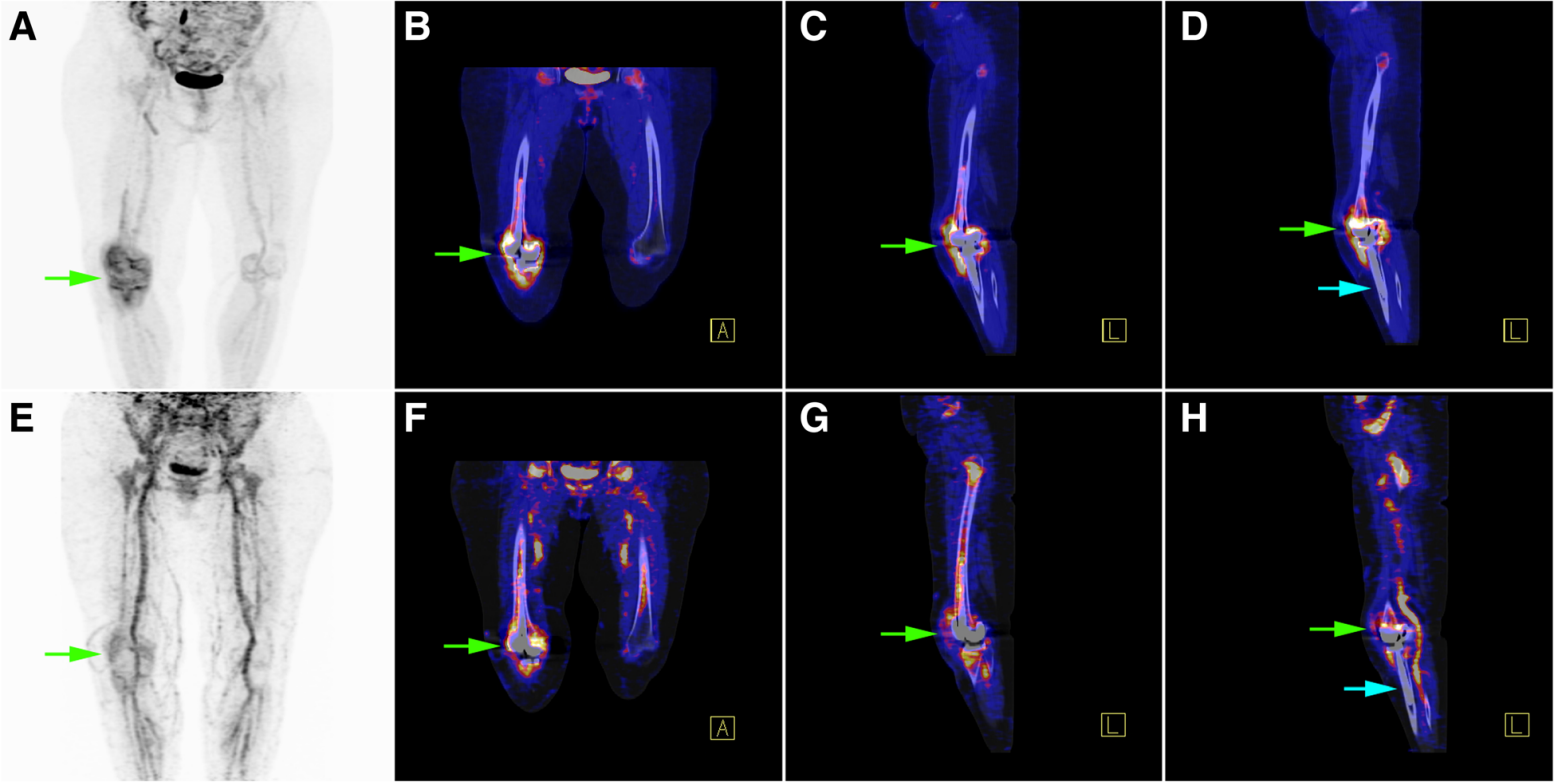
Patient (case # 3) with a history of knee prosthesis implantation performed 12 months before imaging. The results of ^18^F-FDG PET/CT (upper row) and ^68^Ga-citrate PET/CT (lower row) are presented. **a**–**d** The green arrows indicate an increased ^18^F-FDG uptake at both the bone prosthesis interface and the knee synovium. **e**–**h** A similar pattern of ^68^Ga-citrate uptake was evident. The imaging results were considered positive, and the case was subsequently confirmed as true positive. **d**, **h** In contrast to the femoral portion, there was no increased radiotracer uptake along the bone prosthesis interface of the elongated tibial stem component (indicating no loosening or infection of a specific prosthesis segment, blue arrows)

Table 2 depicts the diagnostic characteristics of ^68^Ga-citrate PET/CT and ^18^F-FDG PET/CT scans, which are provided in Figs. 1, 2, 3, and 4. A total of 34 prostheses were examined in the study (19 hip prostheses and 15 knee prostheses). Infections were diagnosed in 26 (76%) patients. According to the radiotracer uptake pattern criteria, the number of true positive, true negative, false positive, false negative patients was 24, 7, 1, 2 for ^68^Ga-citrate PET/CT and 26, 3, 5, 0 for ^18^F-FDG PET/CT, respectively. There were five false positive cases on ^18^F-FDG PET/CT, four of which were considered as true negative on ^68^Ga-citrate PET/CT (cases # 4, 10, 16, and 24). The sensitivity, specificity, and accuracy of ^68^Ga-citrate PET/CT and ^18^F-FDG PET/CT scans were 92%, 88%, and 91% and 100%, 38%, and 85%, respectively (Table 3).

**Table 2:** Diagnostic characteristics of ^68^Ga-citrate PET/CT and ^18^F-FDG PET/CT imaging in detecting infected lower limb prostheses

			^68^Ga-citrate PET/CT				^18^F-FDG PET/CT			
Case #	Site	Surgical results	BPI uptake	Periprosthetic soft tissue uptake	MV	Diagnostic results	BPI uptake	Periprosthetic soft tissue uptake	MV	Diagnostic results
1	RH	+	Yes	Yes	817.00	+ (TP)	Yes	Yes	1212.13	+ (TP)
2	LH	+	No	Yes	576.90	+ (TP)	No	Yes	781.12	+ (TP)
3	RK	+	Yes	No	310.80	+ (TP)	Yes	No	560.66	+ (TP)
4	LH	−	No	No	147.18	− (TN)	No	Yes	469.88	+ (FP)
5	LH	−	No	No	366.66	− (TN)	No	No	402.01	− (TN)
6	LK	+	Yes	No	584.26	+ (TP)	Yes	Yes	955.45	+ (TP)
7	RK	+	Yes	Yes	473.44	+ (TP)	Yes	Yes	666.63	+ (TP)
8	LH	+	Yes	Yes	377.26	+ (TP)	No	Yes	626.08	+ (TP)
9	RK	+	Yes	Yes	595.68	+ (TP)	Yes	Yes	1078.56	+ (TP)
10	LK	−	No	No	338.31	− (TN)	Yes	Yes	434.44	+ (FP)
11	LH	+	Yes	No	579.84	+ (TP)	Yes	No	643.07	+ (TP)
12	LH	−	No	No	327.98	− (TN)	No	No	865.39	− (TN)
13	LH	−	Yes	Yes	424.18	+ (FP)	Yes	Yes	584.04	+ (FP)
14	LH	+	Yes	No	504.58	+ (TP)	Yes	Yes	873.08	+ (TP)
15	LK	+	Yes	Yes	358.36	+ (TP)	Yes	Yes	364.30	+ (TP)
16	RH	−	No	No	173.43	− (TN)	Yes	No	278.18	+ (FP)
17	RH	+	Yes	Yes	348.87	+ (TP)	Yes	Yes	481.64	+ (TP)
18	LK	+	Yes	Yes	376.59	+ (TP)	Yes	Yes	592.65	+ (TP)
19	LH	+	Yes	Yes	477.37	+ (TP)	Yes	Yes	717.30	+ (TP)
20	LH	−	No	No	291.57	− (TN)	No	No	496.38	− (TN)
21	RH	+	Yes	Yes	386.32	+ (TP)	Yes	Yes	611.08	+ (TP)
22	RK	+	Yes	Yes	375.07	+ (TP)	Yes	Yes	542.73	+ (TP)
23	RK	+	Yes	Yes	441.48	+ (TP)	Yes	Yes	514.63	+ (TP)
24	RH	−	No	No	359.94	− (TN)	Yes	Yes	406.57	+ (FP)
25	RH	+	Yes	Yes	273.37	+ (TP)	Yes	Yes	351.80	+ (TP)
26	LH	+	No	No	247.07	− (FN)	No	Yes	259.58	+ (TP)
27	LK	+	Yes	Yes	265.47	+ (TP)	Yes	Yes	325.06	+ (TP)
28	RH	+	Yes	Yes	494.83	+ (TP)	Yes	Yes	533.03	+ (TP)
29	RK	+	Yes	Yes	363.75	+ (TP)	Yes	Yes	546.30	+ (TP)
30	LK	+	Yes	No	309.42	+ (TP)	Yes	Yes	387.73	+ (TP)
31	LK	+	Yes	Yes	266.16	+ (TP)	Yes	Yes	389.33	+ (TP)
32	RK	+	Yes	Yes	492.82	+ (TP)	Yes	Yes	656.04	+ (TP)
33	LK	+	Yes	Yes	297.40	+ (TP)	Yes	Yes	342.49	+ (TP)
34	RK	+	No	No	389.66	− (FN)	Yes	Yes	548.37	+ (TP)

*RH* right hip, *LH* left hip, *RK* right knee, *LK* left knee, *BPI* bone-prosthesis interface, *MV* metabolic volume, *TP* true positive, *TN* true negative, *FP* false positive, *FN* false negative

**Table 3:** Diagnostic performances of ^68^Ga-citrate PET/CT and ^18^F-FDG PET/CT imaging in detecting infected prostheses according to the anatomical site

	Sensitivity (%)	Specificity (%)	Accuracy (%)
Entire cohort (*n* = 34)			
^18^F-FDG PET/CT	100 (26/26)^a^	38 (3/8)^a^	85
^68^Ga-citrate PET/CT	92 (24/26)^a^	88 (7/8)^a^	91
Hip prosthesis (*n* = 19)			
^18^F-FDG PET/CT	100 (12/12)^a^	43 (3/7)^a^	79
^68^Ga-citrate PET/CT	92 (11/12)^a^	86 (6/7)^a^	89
Knee prosthesis (*n* = 15)			
^18^F-FDG PET/CT	100 (14/14)^a^	0 (0/1)^a^	93
^68^Ga-citrate PET/CT	93 (13/14)^a^	100 (1/1)^a^	93

^a^Data in parentheses represent the actual number of cases on which the calculation of sensitivity and specificity was based

**Fig. 5:**
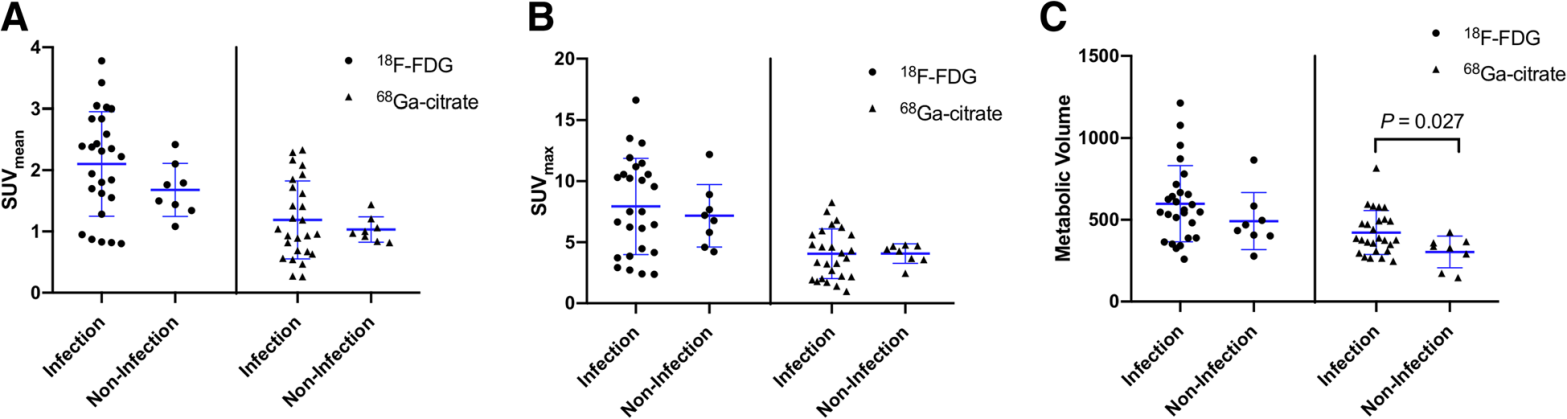
Scatter diagrams for **a** SUV_mean_, **b** SUV_max_, and **c** metabolic volume. Dots and triangles represent ^18^F-FDG PET/CT and ^68^Ga-citrate PET/CT data, respectively. Bars represent mean values ± standard deviations

The median (range) SUV_mean_, SUV_max_, and MV values for the prostheses under examination were 0.99 (0.26–2.33), 4.20 (0.97–8.25), and 370.86 (147.18–817.00) on ^68^Ga-citrate PET/CT scans, respectively, and 1.89 (0.80–3.78), 7.36 (2.38–16.61), and 544.51 (259.58–1212.13) on ^18^F-FDG PET/CT scans, respectively. The SUV_mean_, SUV_max_, and MV values observed on ^18^F-FDG PET/CT images were all significantly higher than those measured on ^68^Ga-citrate PET/CT scans (*p* < 0.001). In ^68^Ga-citrate PET/CT scans, the MV of infected prostheses was significantly higher than that observed for non-infected prostheses (mean ± SD: 422.45 ± 133.87 vs. 303.65 ± 96.39, respectively, *p* = 0.027). However, all of the other measured values did not differ significantly between infected and non-infected prostheses on either ^68^Ga-citrate PET/CT or ^18^F-FDG PET/CT scans (Fig. 5). According to receiver operating characteristic (ROC) curve analysis, a cut-off value of 370.86 for MV resulted in a sensitivity of 61.5% and a specificity of 87.5% (area under curve 0.75, 95% confidence interval 0.57–0.88, *p* = 0.035).

## Discussion

Data on the potential usefulness of ^68^Ga-citrate PET/CT imaging in detecting infected lower limb prostheses remain limited. Although ^18^F-FDG PET has been shown to be highly sensitive in this clinical setting, results on its specificity are less conclusive [18–20]. In the current prospective study, ^68^Ga-citrate PET/CT appeared superior to ^18^F-FDG PET/CT with respect to specificity (88% vs. 38%, respectively), being also successful in distinguishing between an infectious condition and sterile inflammation (Fig. 1).

Non-specific ^18^F-FDG uptake can be persistently detected for several years following an arthroplasty [16]. In a previous experimental study, an increased uptake of both ^18^F-FDG and ^68^Ga was identified in infected bones; however, only ^18^F-FDG accumulation occurred in healing bones free from infections [21]. Owing to different properties and uptake mechanisms for the two radiotracers, we hypothesized that ^68^Ga may be superior to ^18^F-FDG in distinguishing between infectious conditions and non-specific sterile inflammation.

Several mechanisms may potentially explain ^68^Ga-citrate accumulation in infectious foci, including (1) higher Ga extravasation to the extracellular compartment as a result of an increased inflammation-related capillary permeability, (2) binding of dissociated gallium ions to transferring followed by their subsequent sequestration in a highly stable state within an infectious site, (3) binding to bacterial siderophores and activated lactoferrin in neutrophils, and (4) macrophage phagocytosis [22–24]. In contrast, ^18^F-FDG allows the identification of inflammatory cells characterized by an increased glycolysis—ultimately serving as an “in vivo label” for activated inflammatory cells at sites of infection/inflammation [25].

In a previous small cohort study that focused on ^68^Ga-citrate and ^18^F-FDG PET/CT in the detection of infectious foci [26], the use of ^18^F-FDG resulted in a higher target-to-background ratio and higher signals at soft tissue infectious sites. Similar results were observed in the head-to-head comparisons performed in the current study. The SUV_max_, SUV_mean_, and MV values were all significantly higher for ^18^F-FDG PET/CT, which was also superior in diagnosing infections of the sinus tract or cold abscesses. In contrast, ^68^Ga-citrate PET/CT was more effective in identifying the bone remodeling process related to fractures, bone unions, or osteolysis (Figs. 2 and 3).

It is noteworthy that published studies have used different criteria for positive imaging findings, as well as various reference standards [10]. In the current investigation, radiotracer uptake at the bone-prosthesis interface and/or periprosthetic soft tissue was the main criterion for positive results. We cannot exclude that this definition might have contributed to the high sensitivity and low specificity of ^18^F-FDG PET/CT observed in the current study. It should be also kept in mind that adverse reactions to metal debris can result in an increased ^18^F-FDG uptake that is indistinguishable from that occurring at foci of infection [14]. In a previous study that used similar criteria for positive findings, the sensitivity and specificity for infected hip arthroplasties were 100% and 44.8%, respectively [15]. Although the interpretation criteria were identical, the specificity of ^68^Ga-citrate PET/CT was markedly higher than that of ^18^F-FDG PET/CT (88% vs. 38%, respectively). Notably, 20 (58.8%) patients were identified based on the presence of ^68^Ga-citrate uptake in periprosthetic soft tissue. We therefore hypothesize that the high specificity of ^68^Ga-citrate PET/CT compared with ^18^F-FDG PET/CT is not directly linked to a soft tissue uptake underestimation (which should result in a lower likelihood of false positive results) but should rather be ascribed to the intrinsic characteristics of ^68^Ga-citrate. Previous studies have shown that ^18^F-FDG PET may be less accurate in detecting knee prosthesis infections [10, 27]. The question as to whether ^68^Ga-citrate PET/CT imaging can be sufficiently accurate even when the analysis is restricted to knee prostheses alone deserves further scrutiny.

Several authors have maintained that the key to PET/CT diagnosis of prosthesis infections is mainly related to a specific uptake pattern (i.e., radiotracer localization in the bone-prosthesis interface) rather than to the intensity of uptake per se [11, 15]. Our current data lend further support to this prevalent view. Accordingly, SUV_mean_ and SUV_max_ values did not differ significantly between the infected and non-infected groups on both ^18^F-FDG and ^68^Ga-citrate PET/CT imaging. However, we found significant differences in terms of MV on ^68^Ga-citrate PET/CT imaging between infected and non-infected prostheses (422.45 vs. 303.65, respectively, *p* = 0.027). The optimal cut-off value of 370.86 resulted in a sensitivity of 61.5% and specificity of 87.5%. Notably, only two non-infected cases were correctly identifiable beyond the MV range that overlapped with infected cases (Fig. 5). Further studies with larger sample sizes are required to confirm the clinical usefulness of MV quantification on ^68^Ga-citrate PET/CT scans and to further investigate the optimal cut-off value.

Several limitations of our study merit comment. First, our results might not be generalizable owing to the limited sample size and the single-center nature of the study. Second, we did not specifically assess the impact of attenuation-corrected, non-attenuation-corrected, or other different imaging reconstruction methods, which may pose some issues in standalone PET scanners. Third, this study was not designed to evaluate the differences between different interpretation criteria proposed in the literature. Finally, the specificity of ^18^F-FDG PET/CT observed in our study was lower than that previously reported. Consequently, future studies should aim at validating the current data using consistent interpretation criteria and combined PET/CT scanners. Because diagnosis of prosthetic joint infections remains particularly challenging in the early postoperative period [20], we hypothesize that ^68^Ga-citrate PET/CT could serve as a complimentary diagnostic tool especially in patients with recently implanted prostheses.

## Conclusions

Subject to future confirmation, our data provide preliminary evidence that ^68^Ga-citrate PET/CT may have a complimentary role to ^18^F-FDG PET/CT in detecting prosthetic joint infections, being characterized by a higher specificity and the possibility to discriminate between an infectious condition and sterile inflammation.
